# Early-Life Adversity Induces Epigenetically Regulated Changes in Hippocampal Dopaminergic Molecular Pathways

**DOI:** 10.1007/s12035-018-1199-1

**Published:** 2018-09-01

**Authors:** Jana C. Köhler, N. Gröger, A. Lesse, S. Guara Ciurana, K. Rether, J. Fegert, J. Bock, Katharina Braun

**Affiliations:** 1 Department of Zoology/Developmental Neurobiology, Institute of Biology, Otto von Guericke University Magdeburg, Leipziger Straße 44, Bldg. 91, 39120 Magdeburg, Germany; 2 PG “Epigenetics and Structural Plasticity”, Institute of Biology, Otto von Guericke University Magdeburg, Magdeburg, Germany; 3 Klinik für Kinder- und Jugendpsychiatrie/Psychotherapie, Universitätsklinikum Ulm, Ulm, Germany; 4 Center for Behavioral Brain Sciences, Magdeburg, Germany

**Keywords:** Dopamine receptor 1, DARPP-32, Depression, Histone modification, Resilience

## Abstract

Early-life adversity (ELA) represents a major risk factor for the development of behavioral dysfunctions and mental disorders later in life. On the other hand, dependent on type, time point, and duration, ELA exposure can also induce adaptations, which result in better stress coping and resilience later in life. Guided by the hypothesis that chronic exposure to ELA results in dysfunctional brain and behavior, whereas short exposure to ELA may result in resilience, the behavioral and neurobiological consequences of long-term separation stress (LTSS) and short-term separation stress (STSS) were compared in a mouse model for ELA. In line with our hypothesis, we found that LTSS induced depressive-like behavior, whereas STSS reduced depressive-like behavioral symptoms. We then tested the hypothesis that the opposite behavioral outcomes of the two stress paradigms may be mediated by functional, epigenetically regulated changes of dopaminergic modulation in the hippocampal formation. We found that STSS exposure elevated dopamine receptor D1 (DRD1) gene expression and decreased gene expression of its downstream modulator DARPP-32 (32-kDa dopamine- and cAMP-regulated phosphoprotein), which was paralleled by decreased H3 acetylation at its gene promoter region. In contrast, LTSS elevated DARPP-32 gene expression, which was not paralleled by changes in histone acetylation and DRD1 gene expression. These findings indicate that short- and long-term neonatal exposure to ELA induces changes in dopaminergic molecular pathways, some of which are epigenetically regulated and which either alleviate or aggravate depressive-like symptoms later in life.

## Introduction

Early-life adversity (ELA) affects brain development and has been identified as a major risk factor contributing to the etiology of mental disorders, including depression, anxiety and ADHD [1–8]. The particular outcome of ELA exposure has been shown to depend on the type, intensity, and duration of the stressor [9–12]. While the majority of experimental studies focused on stress vulnerability and pathological behavioral outcomes [13–16], evidence is accumulating that acute, brief, or intermittent ELA can promote flexibility and adaptation of behavioral strategies to changing environments [17–20] through activating cellular mechanisms mediating stress resilience [21–24]. So far, the mechanisms determining stress vulnerability or resilience are not well understood. Key mechanisms conferring an individual’s adaptation to environmental challenges include transient or lasting epigenetic alterations as has been shown in a variety of studies in animals and humans [3, 20, 25–30]. Clinical studies revealed that epigenetic modifications are involved in the development of various psychopathologies, such as bipolar disorder and schizophrenia, which are associated with changes in neurotransmitter functions [31, 32]. Since depressive symptoms include anhedonia and despair, dopaminergic modulation of brain regions, which are part of the “reward system,” is particularly affected [33–35]. Animal studies have shown that exposure to ELA as well as variations in maternal care interferes with the maturation of catecholaminergic innervation and dopaminergic function in prefronto-limbic circuits [36–42] and induces sex-specific changes in dopamine receptor D1 (DRD1) density [43] as well as epigenetic modifications regulating the expression of the DRD1 receptor [44, 45]. A key downstream regulator of dopaminergic signaling and mediator of actions and interactions of dopamine with other neurotransmitters is DARPP-32 (32-kDa dopamine- and cAMP-regulated phosphoprotein), which is phosphorylated upon activation of DRD1 receptors. DARPP-32 has been suggested to be involved in neuropsychiatric and neurodegenerative disorders associated with dopaminergic dysfunction [46–49]. For example, it was shown that specific polymorphisms in *PPP1R1B*, the gene encoding DARPP-32 in the human brain, were associated with the risk for schizophrenia [50] and may predict autism susceptibility [51]. Although such polymorphisms are not clearly evident in depression, evidence from human studies indicates a role for DARPP-32 in mood disorders [52]. An animal study described alterations of DARPP-32 in the prefronto-limbic system in mice developing depressive-like symptoms in response to psychosocial stress or social defeat [53, 54].

Based on this evidence, the aims of this study were to compare the behavioral and neurobiological consequences of short-term early-life separation stress (STSS) and long-term separation stress (LTSS) in a mouse model for ELA and to assess changes in DRD1 and DARPP-32 mediated via histone modifications.

## Material and Methods

### Animals

C57BL/6 mice were housed on a 12-h light-dark cycle with food and water provided ad libitum. During pregnancy, the home cages were cleaned once a week. After delivery of the pups (day of birth = postnatal day, PND 0), the home cages were not cleaned for the first 16 postnatal days to minimize stress for the mother and her pups. Males from litters of approximately the same size (five to eight pups) were randomly assigned to the stress groups (see below) and the control groups. The experimental protocols were approved by the ethics committee of the government of the state of Saxony-Anhalt according to the German guidelines for the care and use of animals in laboratory research (§8 TSchG; AZ: 42502–2-1272).

#### Stress Paradigms

##### “Mild” Short-Term Separation Stress (STSS)

Pups of this group were separated from their mother at PND 14–16 using the same separation conditions as described for the LTSS group (see below). After the last separation session on PND 16, the pups remained undisturbed until weaning on PND 21. On PND 21, the animals were reared in groups with a maximum of five individuals until the onset of the experiments.

##### Control (CON) for STSS Paradigm

Animals of this control group lived undisturbed with their mother and littermates. After weaning at PND 21, they were group housed with a maximum of five same-sex individuals until the time of the respective experiment.

##### “Chronic” Long-Term Separation Stress (LTSS)

Pups of this group were exposed to daily maternal separation from PND 1 to PND 21 by removing them from the home cage and individually placed in isolation boxes (13 × 13 cm, covered with paper bedding) for 3 h each day (9:00–12:00), which allowed olfactory and auditory but no visual or body contact with their separated siblings. The dam remained undisturbed in the home cage. Prior to the return of the pups, fresh nesting material was provided. After weaning on PND 21, the animals were housed individually until the time of the respective experiment.

##### Control (CON) for LTSS Paradigm

Animals of this control group lived undisturbed with their mother and littermates. After weaning at PND 21, they were group housed with a maximum of five same-sex individuals until the time of the respective experiment.

### Distribution of Animals

In the STSS experiments, 75 control and 67 stressed animals derived from 24 and 24 litters, respectively, were used. The LTSS experiments included 80 control animals from 24 litters and 50 stressed animals from 20 litters.

### Forced Swim Test

To test for depressive-like behavioral traits, the STSS and their respective control group were subjected to the forced swim test (FST) on PND 62 and 63. On the first day, the animals were habituated to the test situation by transferring them for 15 min to a glass container filled with 22 °C tempered water. On the subsequent test day, the behavior was videotaped during a 15-min trial. Active swimming and passive floating behavior during the first 5 min of the FST were quantified with the “Observer” (Noldus, Wageningen, Netherlands) software. Results for the LTSS group have been published in a previous paper [15].

For the FST in the STSS experiments, 21 control animals and 20 stressed animals, each derived from 6 litters, were used. For the LTSS experiments, 45 control animals derived from 11 litters and 21 stressed animals from 7 litters were used. For the statistical analysis, *N* = number of animals was used.

#### Tissue Preparation

Animals of all experimental and control groups were decapitated on PND 64. Brain tissue of the hippocampal formation was collected and frozen on liquid nitrogen and stored at − 80 °C. Tissue for gene expression and western blot analysis was derived from the same animals, i.e., the hippocampus of the left hemisphere was used for protein extraction, and tissue from the right hippocampus underwent RNA extraction. Tissue for native chromatin immunoprecipitation (nChIP) was derived from additional animals. Hippocampal tissue of the LTSS group from our previous study [15] was collected and analyzed in the same way.

#### Gene Expression

Expression of DRD1 and DARPP-32 mRNA was quantified by real-time quantitative PCR (qPCR). RNA extraction was performed using the RNeasy Mini Kit (Qiagen GmbH, Hilden, Germany). Genomic DNA was removed utilizing the RNase-free DNase Kit (Qiagen GmbH, Hilden, Germany). Gene expression analysis was carried out with the Rotor-Gene Multiplex RT-PCR Kit (Qiagen GmbH, Hilden, Germany), which allows the execution of simultaneous one-step quantitative real-time PCR of two to five genes using TaqMan gene expression assays. Commercially available assays for the dopamine D1 receptor (DRD1; Mm01353211_m1_Drd1a) and the dopamine- and cAMP-regulated neuronal phosphoprotein 32-kDa protein (DARPP-32; Mm00454892_m1_Ppp1r1b) were used. As a reference gene, hypoxanthine phosphoribosyltransferase I (Hprt I; Mm01545399_m1; VIC) was used. Gene expression of DRD1, DARPP-32, and Hprt was calculated utilizing the delta-delta CT method [55]. The samples were normalized to their respective control groups.

For gene expression analysis for the STSS experiments, hippocampal tissue of 41 control animals and 30 stressed animals, each derived from 9 litters, was used, and for the LTSS experiments, 24 control animals derived from 7 litters and 23 stressed animals derived from 6 litters were used. For statistical analysis, *N* = number of animals was used.

#### Histone Acetylation

Acetylation of H3 and H4 was assessed by quantitative western blot (WB) analysis. Tissue samples were homogenized in extraction buffer (0.1 M Tris/HCl pH 8.0; 0.01 M EDTA; 10% SDS; 1× Halt Protease Inhibitor Cocktail (Thermo Fisher Scientific, Waltham, MA, USA)) using ultrasonic vibration. After centrifugation, the supernatant was removed and protein concentration was measured using the Bio-Rad DC™ Universal Protein Assay Kit II (Bio-Rad, Hercules, CA, USA). SDS-PAGE was performed using a 20-μl reaction volume per lane, which contained 50 μg protein mixed with loading buffer and Bio-Rad Tris-Glycine Mini-PROTEAN TGX Precast Gels (Bio-Rad, Hercules, CA, USA). The prestained protein molecular weight marker PeqGOLD Protein Marker V (PeqLab/VWR International GmbH, Darmstadt, Germany) was used to monitor the progress of SDS-PAGE and to assess transfer efficiency onto the membrane during western blot. After gel electrophoresis, the samples were blotted onto a nitrocellulose membrane (GE Healthcare, Chalfont St Giles, UK), and the proteins were visualized by Red Alert staining (Novagen, brand by Merck KGaA, Darmstadt, Germany). The blots were blocked by using Roti-Block (Carl Roth, Karlsruhe, Germany) working solution followed by overnight incubation with primary antibodies anti-acetyl H3 (#06-599, 1:10,000; Merck Millipore, Billerica, MA, USA), anti-acetyl H4 (#06-866, 1:4000; Merck Millipore, Billerica, MA, USA), and antihypoxanthine phosphoribosyltransferase I (#ab10479, 1:500; Abcam, Cambridge, UK) at 4 °C. The blots were then incubated for 1 h at room temperature with a secondary antibody (horseradish peroxidase conjugated anti-rabbit) (#12-348, 1:4000; Novagen, brand by Merck Millipore, Billerica, MA, USA). Horseradish peroxidase was detected by using Luminata Crescendo Western HRP substrate (Merck Millipore, Billerica, MA, USA), and signals were detected by using Syngene G:Box system (Syngene Europe, Cambridge, UK). Western blot data were analyzed using Gene Tools software (Syngene Europe, Cambridge, UK).

Histone acetylation analysis for the STSS experiments was conducted in hippocampal tissue from 48 control animals and 41 stressed animals, each derived from 12 litters. For the LTSS experiments, 29 control animals and 23 stressed animals, each derived from 7 litters, were used. For statistical analysis, *N* = number of litters.

#### Histone Acetylation at the DARPP-32 Promoter

H3 and H4 acetylation associated to DARPP-32 expression was quantified by nChIP. Tissue samples were homogenized in 500 μl MNase Reaction Buffer (10 mM Tris pH 8.8, 1 mM CaCl_2_). After adding 10 units MNase (Affymetrix, Cleveland, OH, USA), the samples were incubated for 2 min at 37 °C. The enzymatic reaction was blocked by adding 10 μl 0.5 M EDTA. The samples were mixed with 5 ml 0.02 mM EDTA and 6 μl 100 mM PMSF and were incubated for 1 h on ice while mixing them every 10 min. After the incubation period, 9 μl 1 M DTT was added and the samples were centrifuged at 4000×*g* for 10 min at 4 °C. The supernatant was divided into four parts: 500 μl input and 3 × 1500 μl H3, H4, and control. The three latter samples (H3, H4, control) were incubated for 2 h at room temperature with the respective antibodies: anti-acetyl H3 (#06-599, 1:10,000; Merck Millipore, Billerica, MA, USA) and anti-acetyl H4 (#06-866, 1:4000; Merck Millipore, Billerica, MA, USA). Protein A/G Magnetic Beads (Thermo Fisher Scientific, Waltham, MA, USA) were used for immunoprecipitation. Before use, the beads were washed twice with TBS wash buffer containing 0.05% Tween-20 and 0.5 M NaCl. The beads and the ChIP-antibody samples were incubated overnight at 4 °C while rotating. Beads were separated from unbound sample fraction and washed twice using TBS wash buffer and once with dH_2_O. The DNA was then eluted in 100 μl low pH elution buffer (0.1 M glycine pH 2.0). After incubating for 10 min, the eluates were separated from the beads and pH was neutralized with 15 μl 1 M Tris pH 7.5. Subsequently, the samples were subjected to protein digestion by adding 400 μl Tris-HCl pH 7.5, 20 μl Tris-HCl pH 6.8, 20 μl 5 M NaCl, 10 μl 0.5 mM EDTA pH 8.0, and 1 μl 20 mg/ml Proteinase K (Roche Diagnostics, Rotkreuz, Switzerland) and incubated overnight.

DNA was extracted from the sample by adding 500 μl phenol/chloroform/isoamylalcohol (Carl Roth GmbH, Karlsruhe, Germany). DNA purification was performed using the MinElute Reaction CleanUp Kit (Qiagen GmbH, Hilden, Germany). ChIP quantitative real-time PCR was carried out using the Rotor-Gene Multiplex PCR Kit (Qiagen, Hilden, Germany). A custom-made FAM-coupled TaqMan assay targeting the DARPP-32 promoter region was used (Life Technologies, Carlsbad, CA, USA).

In both the STSS and the LTSS experiments, histone acetylation at the DARPP-32 promoter was analyzed in hippocampal tissue from six control animals and six stressed animals, each derived from six litters. For statistical analysis, *N* = number of animals was used.

### Statistical Analyses

The data for each experiment were tested for normal distribution using the D’Agostino and Pearson omnibus normality test. If normally distributed, data were analyzed for significance with a two-sided unpaired *t* test. Non-normally distributed data were tested for significance with a Mann-Whitney *U* test. Data of the gene expression analysis were normalized to controls. All data are presented as means ± SEM. Significance was set at *p ≤ 0.05 and **p ≤ 0.01 for all data sets. Statistical analysis was performed and graphs were produced using GraphPad Prism 6.0 software (GraphPad, La Jolla, CA, USA).

## Results

### Behavior

STSS resulted in reduced depressive-like behavior in adulthood, measured as lower time of immobility (floating) compared to controls (*p* = 0.005, Table 1; *N*: CON = 21, STSS = 20). In contrast, LTSS (PND 1–21) induced enhanced depressive-like behavior measured as elevated duration of immobility as published in a previous study [15].

**Table 1:** Duration of immobility in the forced swim test

Treatment		*N*	Duration of immobility (mean ± SEM) (s)	*p* value
STSS	Stressed	20	68.67 ± 9.76	0.005
	Control	21	124.1 ± 14.7	
LTSS	Stressed	21	195.6 ± 10.69^a^	0.015
	Control	45	161.8 ± 7.73^a^	

^a^Published in [15]

### Gene Expression

**Fig. 1:**
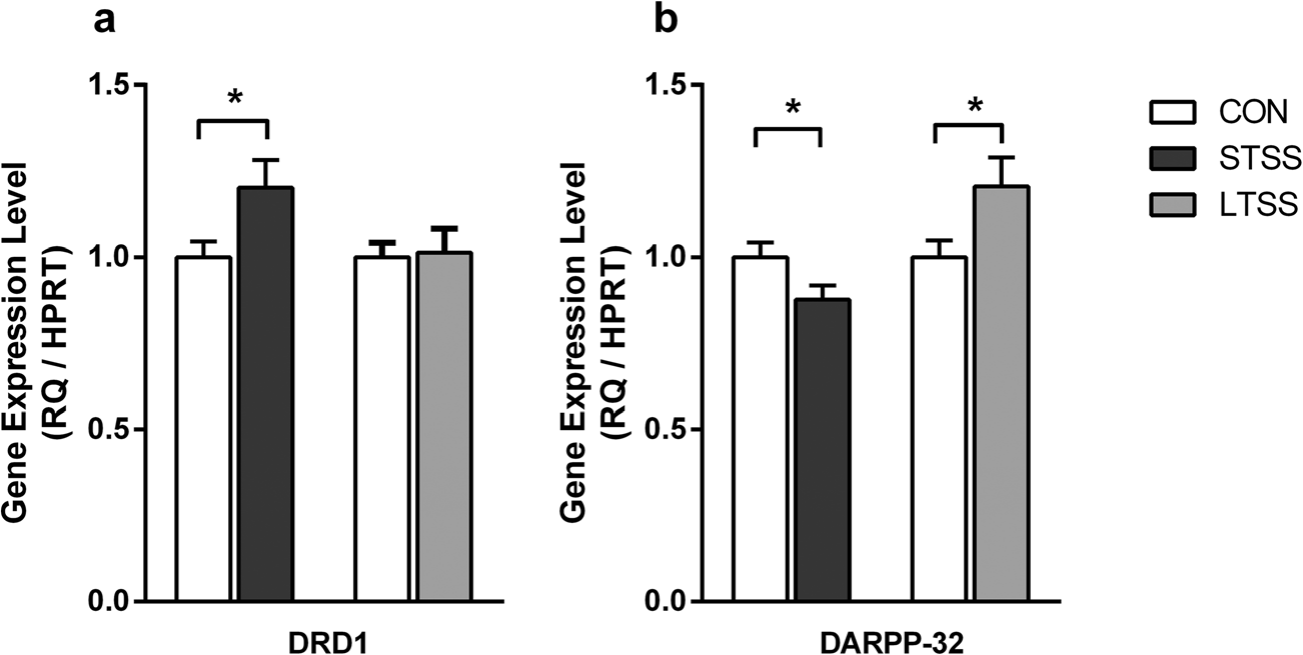
ELA induces changes in DRD1 and DARPP-32 gene expression. **a** Short-term separation stress (STSS) induced an increase in DRD1 gene expression, whereas long-term separation stress (LTSS) had no effect on the expression of the DRD1 gene (*p ≤ 0.05). **b** STSS induced reduced gene expression of DARPP-32; in contrast, LTSS induced increased DARPP-32 expression (*p ≤ 0.05)

In the hippocampus of the STSS group, increased DRD1 expression was found compared to the control group (*p* = 0.023, Fig. 1a; *N*: CON = 39, STSS = 28), whereas in LTSS animals, DRD1 expression remained unchanged compared to controls (*p* = 0.433, Fig. 1a *N*: CON = 24, LTSS = 22). Expression of DARPP-32 was decreased in the STSS group (*p* = 0.049, Fig. 1b; *N*: CON = 41, STSS = 30), whereas it was increased in the LTSS group (*p* = 0.033, Fig. 1b; *N*: CON = 24, LTSS = 22).

### Histone Acetylation

**Fig. 2:**
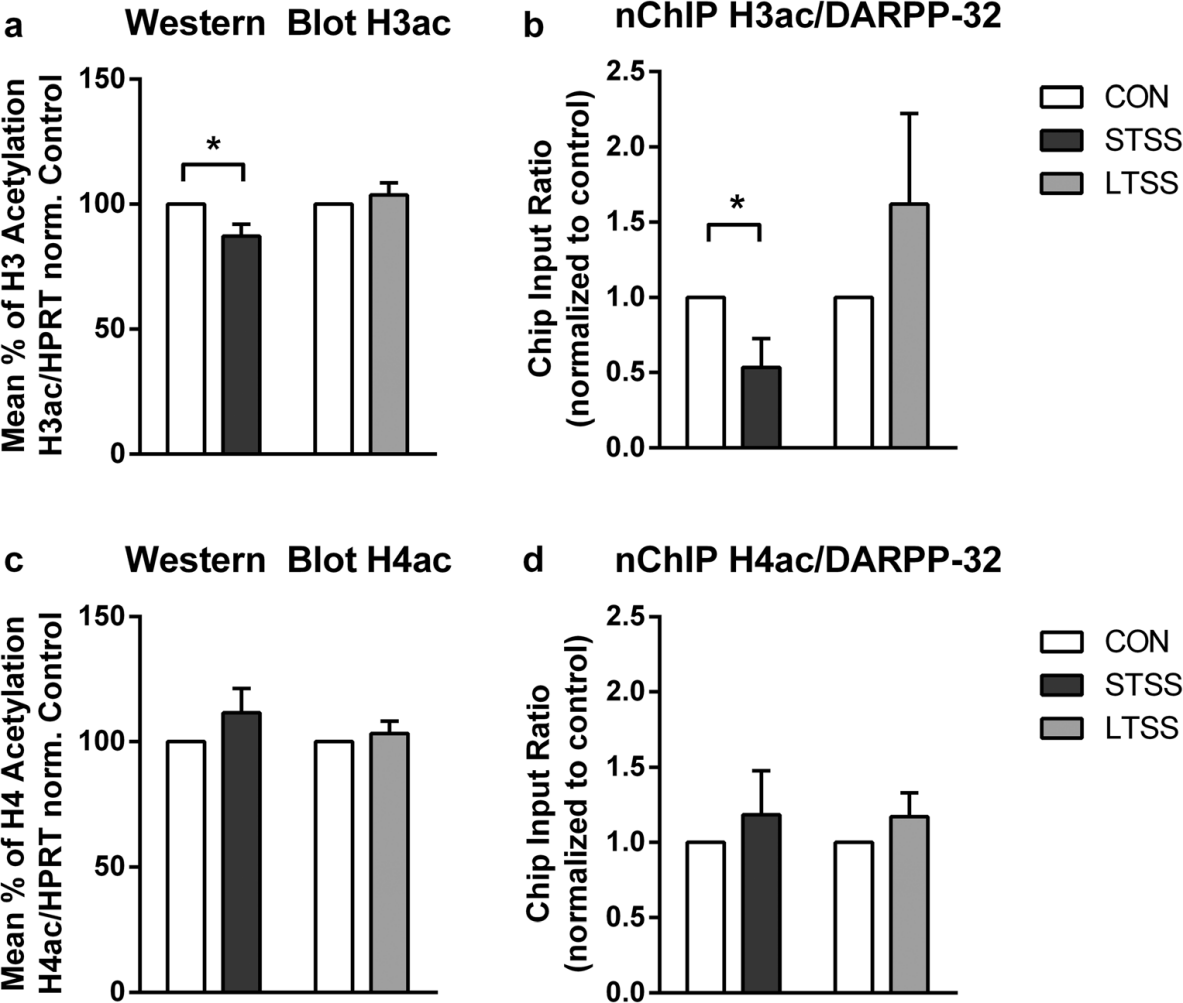
ELA-induced changes in histone acetylation. **a** STSS induced a reduction of H3 acetylation in the hippocampus, whereas LTSS had no effect (*p ≤ 0.05). **b** STSS resulted in reduced H3 acetylation at the promoter region of DARPP-32, whereas LTSS had no effect (*p ≤ 0.05). **c**, **d** Global H4 acetylation as well as DARPP-32 promoter-specific H4 acetylation was affected neither by STSS nor by LTSS

STSS decreased histone H3 acetylation in the hippocampus compared to control animals (*p* = 0.0147, Fig. 2a; *N*: CON = 11, STSS = 11), and no significant changes were detected in histone H4 acetylation (*p* = 0.248, Fig. 2c; *N*: CON = 12, STSS = 12). LTSS did not affect histone H3 and H4 acetylation (*p* = 0.459, Fig. 2a; and *p* = 0.497, Fig. 2c; *N*: CON = 7, LTSS = 7).

#### Histone Acetylation at the DARPP-32 Promoter

nChIP-qPCR revealed that the reduced expression of DARPP-32 was associated with reduced acetylation of H3 at the promoter region of its gene (*p* = 0.0252, Fig. 2b; *N*: CON = 6, STSS = 5), while H4 acetylation at the promoter region of DARPP-32 was not altered (*p* = 0.5414, Fig. 2d; *N*: CON = 6, STSS = 6). In the LTSS group, H3 and H4 acetylation at the DARPP-32 promoter region was unchanged (*p* = 0.285, Fig. 2b; *p* = 0.264, Fig. 2d; *N*: CON = 7, LTSS = 7).

## Discussion

In line with our working hypothesis, we show that exposure to “mild” STSS reduces depressive-like behavioral symptoms in adulthood, whereas a previous study [15] demonstrated that “chronic” LTSS increased depressive-like symptoms. We further found that the opposing behavioral outcomes were paralleled by partly opposite changes in dopaminergic cellular pathways in the hippocampal formation. STSS elevated DRD1 gene expression and decreased gene expression of its downstream regulator DARPP-32, the latter of which is regulated via reduced H3 acetylation at its promoter region. In contrast, LTSS did not alter DRD1 gene expression and elevated DARPP-32 gene expression, which was not associated with changes in histone acetylation. Global hippocampal histone H3 acetylation was reduced after STSS but not after LTSS.

### Methodological Considerations

With respect to our STSS paradigm, it is important to point out that this manipulation not only represents a mild, short-term stress experience but is also restricted to a defined developmental time window, i.e., after the end of the so-called stress hyporesponsive period (SHRP) of the HPA axis. In mice and rats, the SHRP is characterized by low levels of circulating corticosterone and a relative hyporesponsiveness to mild environmental stressors [56, 57]. Our previous studies in rats revealed that the extent and direction of stress-induced neuromorphological outcomes are dependent on the time point of the respective stress exposure (maternal separation), i.e., prior, during, or after the SHRP [58, 59]. We specifically selected a time window after the SHRP for this experiment since our previous study in mice demonstrated that stress exposure during this time window increases dendritic complexity and the number of excitatory spine synapses of hippocampal CA3 neurons [19], which we predicted to reflect positive behavioral outcome. Comparing the behavioral assessment during the FST, it is obvious that there are significant differences between the immobility times of the two separate control groups, i.e., the control group for the present study and the control group for the previous study [15]. The animals of the respective control groups were derived from completely different cohorts, and it is not unusual that basal behavioral parameters may differ between such separate samples. Hence, this finding emphasizes the importance of running parallel control animals for every experiment so that they can be internally compared to the respective experimental group. With respect to our findings, the difference between the two separate control groups does not affect the results, since the STSS animals show lower immobility levels than their respective control group and the LTSS animals display higher levels than their respective control group.

### ELA-Induced Changes of Depressive-like Behavior Depend on Chronicity of Stress Experience

As stated above, the results of the present and previous studies [15] revealed opposite behavioral outcomes. First of all, it is important to state that due to the large spectrum of different maternal separation (MS) paradigms (procedure, timing, duration, etc.) used by different research groups, it is difficult to compare the specific outcomes that have been described [60–62]. Various studies have reported an increase in depressive-like behavior as a consequence of repeated MS [63–67], whereas evidence is accumulating from clinical and animal studies that stress experience early in life may also promote adaptive effects that are beneficial to emotional and cognitive development. Rats exposed to MS displayed reduced immobility time in the FST [68], and a combination of MS with reduced bedding material (maternal neglect paradigm) increased active behaviors in the FST [69]. A recent study in mice revealed that exposure to ELA induced by limited nesting and bedding material leads to better coping with challenging environments in adulthood [18]. These findings can be interpreted within the concept of the “stress-inoculation/induced resilience” hypothesis [70–81]. Along the same line, the present study revealed that STSS results in reduced “behavioral despair” symptoms (less floating), indicating better stress coping in this group, in contrast to animals exposed to LTSS, which displayed increased “behavioral despair” [15].

### ELA “Reprograms” Dopaminergic System Development

The dopaminergic system is involved in the modulation of a number of important brain functions such as motor control, control of emotional behavior, reward, cognition, and decision-making. Consequently, dysfunctions in the dopaminergic system are implicated in a number of pathologies such as Parkinson’s disease, schizophrenia, depression, and ADHD. Studies in a variety of species and stress paradigms revealed that the development of the dopaminergic system is particularly sensitive toward ELA such as prenatal stress [4, 25] and neonatal stress experience [26, 36, 40, 43, 82–84]. With regard to changes of dopamine receptors, it has been reported in rats that MS (24 h on PND 9) increases the expression of DRD1 (and DRD2) receptors [16, 85]. Similarly, a study in male mandarin voles reports that early deprivation leads to an increase of D1 and D2 receptors [86]. Evidence for dysfunctional development of the dopaminergic system is also revealed by a series of studies in the biparental rodent *Octodon degus*, which show that exposure to repeated brief parental separation stress induces an increase in DRD1 receptor (and other receptors) expression in the hippocampal formation [43] and the amygdala (only in females). In addition, chronic, repeated exposure to parental separation resulted in decreased dopaminergic innervation in the hilus of the dentate gyrus and increased innervation in the stratum granulosum and subgranular layer, and changes in dopaminergic innervation were also observed in prefrontal and other limbic regions [38, 40, 87].

The reduced “behavioral despair” symptoms observed in STSS animals during the FST might be mediated by elevated expression of DRD1 in the hippocampal formation. This view is supported by pharmacological analyses in mice, which revealed that passive behavioral traits in the forced swim test induced by MS are specifically mediated by DRD1 [63]. Dopamine D1 receptor agonists were found to induce anti-immobility effects, and the “antidepressant” effect of imipramine can be antagonized by SCH 23390, a selective dopamine D1 receptor blocker [88, 89].

Exposure to STSS resulted in a downregulation of DARPP-32, a key mediator of dopaminergic transmission [46, 49, 90], whereas it was upregulated in the LTSS group. It is tempting to speculate that changes in hippocampal DARPP-32 gene expression may alter short-term and long-term hippocampal synaptic plasticity. DARPP-32 is required for LTP induction in the hippocampal-PFC pathway [91], and DRD1 activation is involved in late LTP [92–94]. Moreover, it was reported that LTP induction in hippocampal afferents into the PFC, which depends on DRD1 activation [95], was facilitated after short stress exposure, whereas prolonged exposure to stress impaired LTP induction [96].

### ELA-Induced Downregulation of DARPP-32 Expression Is Mediated by Specific Epigenetic Histone Modification

First, the present study revealed a global reduction in H3 acetylation in the STSS but not in the LTSS group, which is in line with the concept that gene × environment interactions are mediated by specific epigenetic modifications. With respect to ELA, a number of studies revealed an influence on epigenetic mechanisms that interfere with preprogrammed developmental processes resulting in adaptive or maladaptive neuronal and behavioral alterations [4, 97–104]. Epigenetic adaptations in response to early environmental challenges are dynamic multistep events starting with rapid and transient alterations, some of which eventually may become permanent epigenetic marks [75, 105]*.* Our results are in line with this dynamic concept as we observed a rapid increase in H3 as well as H4 acetylation in the hippocampus of juvenile STSS-exposed animals [19], while the present study demonstrates a lasting reduction of hippocampal H3 acetylation in adult STSS-exposed animals.

Second, we also show that the STSS-induced reduction in DARPP-32 gene expression is mediated by a reduction of H3 acetylation at the promoter region of the DARPP-32 gene, whereas elevation of DARPP-32 gene expression observed in the LTSS group appears not to be related to histone modifications; hence, it remains to be further investigated whether other epigenetic mechanisms, such as DNA methylation, may be involved.

In conclusion, this study revealed differential behavioral, molecular, and epigenetic changes in response to exposure to “mild” STSS and chronic LTSS, which are potentially related to improved (STSS exposure) or impaired (LTSS exposure) adaptability and coping toward environmental adversities later in life.
